# Reduced Fat Taste Sensitivity and Its Association with Childhood Obesity in Tunisian Children: A Cross-Sectional Study

**DOI:** 10.3390/nu17193095

**Published:** 2025-09-29

**Authors:** Rym Ben Othman, Inchirah Karmous, Farah Aissa, Halil İbrahim Ceylan, Youssef Zanina, Henda Jamoussi, Nicola Luigi Bragazzi, Ismail Dergaa

**Affiliations:** 1 National Institute of Nutrition and Food Technology of Tunis, 11 Jbal Lakhdar Street, Tunis 1007, Tunisia; benothmanr@gmail.com (R.B.O.); hendajamoussi@gmail.com (H.J.); 2 Faculty of Medicine of Tunis, University of Tunis El Manar, 13 Jbal Lakhdhar Street, Tunis 1007, Tunisia; 3 Research Unit of Obesity: Etiopathogenesis, Pathophysiology and Treatment (UR18ES01), Tunis 1007, Tunisia; inchirah.karmous@yahoo.fr; 4 Higher School of Health Sciences and Techniques of Tunis, University of Tunis El Manar, 11 Djebel Lakhdhar Street, Bab Saadoun, Tunis 1007, Tunisia; farahissa648@gmail.com; 5 Department of Physical Education of Sports Teaching, Faculty of Sports Sciences, Atatürk University, Erzurum 25240, Turkey; 6 Faculty of Pharmacy of Monastir, University of Monastir, Monastir 5000, Tunisia; zanina.youssef@protonmail.com; 7 Laboratory for Industrial and Applied Mathematics (LIAM), Department of Mathematics and Statistics, York University, Toronto, ON M3J 1P3, Canada; 8 High Institute of Sport and Physical Education of Ksar Said, University of Manouba, Manouba 2010, Tunisia; phd.dergaa@gmail.com; 9 Physical Activity Research Unit, Sport and Health (UR18JS01), National Observatory of Sports, Tunis 1003, Tunisia

**Keywords:** childhood obesity, fat taste sensitivity, gustatory perception, linoleic acid, nutritional assessment, pediatric health, sensory evaluation, sweet taste threshold

## Abstract

Background: Childhood obesity is a growing public health challenge, with altered taste perception potentially influencing food choices and contributing to weight gain. Objective: To determine detection thresholds for linoleic acid (fat taste) and sucrose (sweet taste) in children aged 6–12 years, and to explore associations with obesity, dietary intake, and food preferences. Methods: In this cross-sectional study, 100 Tunisian children (mean age: 8.05 ± 1.44 years; 54% girls; 45 obese, 55 non-obese) were recruited from an educational support center in Nabeul. Taste sensitivity was evaluated using the 3-alternative forced choice (3-AFC) method with ascending concentrations of linoleic acid (0.018–12.0 mM) for fat taste and sucrose (0.00125–0.32 mol/L) for sweet taste. Participants were categorized as tasters or non-tasters based on detection thresholds. Anthropometric measurements, 24 h dietary recalls, food frequency questionnaires, and food preference assessments were also conducted. Results: Low taste sensitivity was common (93% for sweet, 49% for fat). Girls were more often fat tasters than boys (68.6% vs. 31.4%, *p* = 0.003). Children with obesity had higher fat taste thresholds (median 3.00 mM, range 0.37–12.0) than non-obese peers (median 1.50 mM, range 0.018–6.0; *p* = 0.012), indicating reduced fat taste sensitivity. Linear regression showed a significant positive association between fat taste threshold and BMI (*p* = 0.001), meaning higher detection thresholds corresponded to higher BMI. Sweet taste thresholds did not differ significantly between children with and without obesity (*p* = 0.731). Sweet non-tasters consumed more sucrose (85.9 ± 64.9 g/d vs. 70.3 ± 62.3 g/d; *p* = 0.033) and reported more frequent table sugar use (*p* = 0.047). Fat non-tasters consumed more magnesium (425 ± 414 mg/d vs. 287 ± 60.8 mg/d; *p* = 0.026) and fiber (22.9 ± 7.51 g/d vs. 20.3 ± 5.32 g/d; *p* = 0.048) and reported higher intake frequencies of cheese (*p* = 0.039), sour cream (*p* = 0.004), and fast food (*p* = 0.012). Food preferences reflected similar patterns, with non-tasters generally rating high-fat or high-sugar foods more favorably. While most children demonstrated high detection thresholds, girls showed significantly higher fat taste sensitivity compared to boys (*p* = 0.03). Children with obesity exhibited significantly higher fat taste detection thresholds compared to non-obese children (*p* = 0.012), with thresholds ranging from 0.37 to 12.0 mM versus 0.018 to 6.0 mM, respectively. No significant difference was observed for sweet taste perception between weight groups (*p* = 0.731). Conclusions: Nearly half of the children exhibited reduced fat taste sensitivity, which was moderately associated with obesity and positively linked to BMI.

## 1. Introduction

Childhood obesity has become one of the most significant global health crises of our time, with countless children and families worldwide experiencing its effects as rates continue to rise across diverse populations and communities [1]. Current epidemiological data from the Global Burden of Disease Study 2021 indicate that worldwide obesity in children and adolescents has continued to rise substantially, with projections showing continued increases through 2050 if current trends persist [2]. This alarming trend carries profound implications for healthcare systems, with children with obesity facing increased risks of developing type 2 diabetes, cardiovascular disease, and metabolic syndrome during childhood and adulthood [3]. The economic burden associated with childhood obesity is substantial, affecting not only direct healthcare costs but also imposing significant societal costs through reduced quality of life and decreased productivity [4]. Beyond immediate health consequences, childhood obesity frequently persists into adulthood, with the majority of adolescents with obesity remaining adults with obesity, thereby perpetuating a cycle of chronic disease and healthcare utilization [5].

The pathophysiology of childhood obesity involves complex interactions between genetic predisposition, environmental factors, and behavioral patterns, with taste perception playing an increasingly recognized role in dietary choice and food intake regulation [6,7]. Taste sensitivity, particularly for sweet and fat tastes, represents a fundamental component of appetite regulation and food preference development, with genetic variations in taste receptors significantly influencing individual sensitivity patterns [8,9,10]. The sweet taste is instinctively appreciated from birth, serving as an evolutionary mechanism to identify energy-rich foods. In contrast, fat taste perception involves the Cluster of Differentiation (CD36) receptor, also known as fatty acid translocase, and influences food palatability through textural enhancement and satiety signaling [11,12].

During childhood, taste preferences undergo significant developmental changes, with early exposure patterns having a strong influence on long-term dietary behaviors and weight outcomes [13,14]. Chronic exposure to high-sugar and high-fat foods can lead to sensory adaptation, requiring increasingly higher concentrations to achieve equivalent taste satisfaction, which may promote overconsumption patterns [15]. Furthermore, obesity-related inflammatory processes may directly affect taste bud function and regeneration, creating a bidirectional relationship between weight status and gustatory perception [16,17].

Despite growing recognition of the importance of taste perception in the development of childhood obesity, significant research gaps persist in our understanding of these mechanisms. First, limited data exist regarding taste sensitivity thresholds in pediatric populations [18,19], with most studies focusing on adult participants or specific clinical populations such as pregnant women with diabetes [6,7,20,21,22]. Second, substantial variations in taste perception methodology and threshold definitions across studies make cross-study comparisons difficult and limit the development of standardized assessment protocols [7]. Third, the lack of data from developing countries and non-Western populations limits our understanding of how cultural dietary patterns and genetic variations influence the development of taste sensitivity [13,23]. Fourth, robust statistical analyses examining independent risk factors for altered taste perception in children with obesity versus children without obesity remain limited, hindering identification of modifiable intervention targets [24,25]. Fifth, incomplete characterization of the relationship between taste sensitivity and actual dietary intake patterns limits our ability to develop evidence-based nutritional counseling strategies [14,26]. Sixth, lack of validated, cost-effective screening tools for clinical assessment of taste sensitivity in pediatric settings prevents integration of gustatory evaluation into routine obesity management protocols [27,28].

Based on these research gaps, this study aimed to determine the detection thresholds for linoleic acid and sucrose in children aged 6 to 12 years using a standardized 3-alternative forced-choice methodology. Sucrose was chosen as the prototypical sweet tastant due to its innate preference from birth, ubiquity in children’s diets, and established role in driving excessive sugar intake and increasing obesity risk. Linoleic acid was selected as the reference fatty acid stimulus due to its physiological relevance as an essential nutrient, its role as a ligand of the CD36 receptor mediating fat taste perception, and its abundance in commonly consumed oils and processed foods. Secondary objectives included examining the association between taste sensitivity and childhood obesity, evaluating the relationship between taste perception and dietary preferences, and identifying demographic and anthropometric factors associated with altered gustatory function in this pediatric population.

## 2. Materials and Methods

### 2.1. Ethical Approval

This study received approval from the Ethics Committee of the National Institute of Nutrition and Food Technology of Tunis (No. 19/2023) and was conducted in accordance with the principles of the Declaration of Helsinki for research involving human subjects. Written informed consent was obtained from all parents or legal guardians after a comprehensive explanation of the study objectives, procedures, potential risks, and benefits. Children provided verbal assent before participation. All participants retained the right to withdraw from the study at any time without penalty or explanation.

### 2.2. Study Design

This cross-sectional observational study was conducted from November 2024 to February 2025 in Nabeul, Tunisia. The study employed standardized sensory evaluation protocols to assess taste perception thresholds and examine associations with anthropometric, dietary, and demographic variables in a pediatric population.

### 2.3. Sample Size Calculation

Sample size estimation was based on previously published data examining taste threshold differences between children with and without obesity [23]. Using a mean taste threshold of 18.49 (standard deviation, SD = 8.53) for normal-weight children versus 19.33 (SD = 9.75) for children with obesity, the pooled SD was calculated as 9.16, yielding a small effect size (Cohen’s d) of approximately 0.092. To detect this difference with 80% power and α = 0.05 (two-tailed test), an estimated sample size of 862 participants per group would be required. Given the small effect size, we implemented a binary classification system (“tasters” versus “non-tasters”) to reduce the sample size needed. With no previous studies employing this binary classification for detecting fat and sweet tastes in children, we proceeded with a convenience sample of 45 children with obesity and 55 children without obesity, aged 6–12 years.

### 2.4. Participants

Children were recruited from an educational support center in Nabeul, Tunisia, using convenience sampling methodology. Inclusion criteria included: age between 6 and 12 years, apparently healthy status with no acute illnesses, and parental consent with child assent. Exclusion criteria encompassed: type 1 or type 2 diabetes mellitus, oral diseases or dental pathology affecting taste perception, recent flu or cold symptoms, acute infections within 2 weeks before testing, and use of medications known to affect taste sensation.

### 2.5. Study Procedures

Since this study involved questionnaire administration, we ensured the highest standards in applying psychometric methods throughout the entire study protocol, as highlighted by Guelmami et al. [29].

Linoleic acid (Free Acid, extrapure, 98%) was purchased from ABTECH (Le Kram, Tunisia; catalogue NO. 98099). It was stored at −20 °C in amber, nitrogen-flushed vials until use to minimize oxidation. To reduce adsorption, we used silanized amber vials with Teflon-lined caps for storage and handling. All procedures were performed under subdued light and with minimal air exposure.

A concentrated stock solution of linoleic acid (100 mg·mL^−1^) was first prepared in absolute ethanol. The aqueous phase consisted of Milli-Q water containing 2% (*w*/*v*) gum arabic, which served as an emulsifying agent. The linoleic acid stock was added dropwise to the aqueous phase under continuous magnetic stirring at 700 rpm, resulting in the final emulsion concentrations.

Coarse emulsification was performed with a high-shear homogenizer at 12,000 rpm for 2 min on an ice bath to prevent heating. Droplet size was further reduced by probe sonication at 30% amplitude in pulsed mode (3 × 30 s on/30 s off), with the sample tube immersed in ice to maintain temperature below 10 °C. After sonication, emulsions were equilibrated for 10 min at 4 °C and inspected for phase separation.

To minimize oxidation of polyunsaturated fatty acids, all emulsions were prepared under a gentle stream of nitrogen and transferred into amber glass vials with Teflon-lined caps. Fresh emulsions were used within 48 h of preparation. To limit oxidative degradation, all steps that could influence oxidation or adsorption (such as exposure to air, elevated temperature, or prolonged light exposure) were minimized. Personnel wore nitrile gloves and used dedicated pipettes and glassware for handling fatty acids.

Taste sensitivity evaluation employed the 3-alternative forced choice (3-AFC) method, a validated psychophysical technique for threshold determination. Solutions were prepared a maximum of 48 h before testing in the laboratory of the National Institute of Nutrition and Food Technology of Tunis under standardized conditions. All participants fasted for a minimum of 2 h before testing to prevent interference with taste sensitivity assessment.

For fat taste evaluation, linoleic acid solutions were prepared at concentrations ranging from 0.018 to 12.0 mM using 5% gum arabic as a carrier solution. Control solutions contained only water with 5% gum arabic. The fat samples were prepared by dissolving linoleic acid in a 5% gum Arabic solution, followed by thorough homogenization under controlled conditions, and freshly prepared aliquots were used for testing to ensure stability and reproducibility. Participants received three glasses per concentration level: two containing the control solution and one containing the test concentration. Testing progressed from lowest to highest concentration until participants correctly identified the “oily taste” in three consecutive trials, establishing the recognition threshold.

Sweet taste assessment utilized sucrose solutions ranging from 0.00125 to 0.32 mol/L prepared by serial dilution from a stock solution (6.4 × 10^−1^ mol/L). Control solutions contained water only. A similar 3-AFC methodology was employed, with progression through increasing concentrations until participants correctly detected “sweet taste” in three consecutive presentations.

Participants were classified as “tasters” (able to detect taste at any concentration) or “non-tasters” (unable to detect taste regardless of concentration). Tasters were further subdivided into “super tasters” (recognition at low concentrations) and “low tasters” (recognition only at high concentrations) based on established threshold categories (Table 1 and Table 2).

### 2.6. Anthropometric Measurements

Height and weight were measured using calibrated equipment following standardized protocols. Body mass index (BMI) was calculated as weight (kg) divided by height squared (m^2^). BMI classification employed age- and sex-specific percentile curves based on World Health Organization growth standards [30]. Normal weight was defined as BMI between the 15th and 85th percentiles, while obesity was defined as BMI above the 97th percentile.

### 2.7. Dietary Assessment

Dietary intake was evaluated using 24 h nutritional recall methodology, coupled with an assessment of habitual dietary pattern, administered by trained personnel. Food quantities were estimated using a standardized photographic manual. Nutritional analysis was performed using NUTRILOG Online software, providing comprehensive macronutrient and micronutrient intake data, including energy, carbohydrates, proteins, lipids, vitamins, minerals, and fiber content.

Food frequency questionnaires assessed consumption patterns for specific food categories. Additionally, food preference questionnaires required children to rate their attraction to various foods on a 0–10 scale, providing a quantitative assessment of dietary preferences. Data collection was carried out through structured interviews with the children, with parents in charge of delivering or confirming responses when necessary.

### 2.8. Statistical Analysis

Data analysis was performed using GraphPad Prism 9.0 and SPSS 27.0 software. Normality of continuous variables was assessed using the Kolmogorov–Smirnov test. Descriptive statistics were expressed as mean ± SD for normally distributed variables and median (25th–75th percentile) for non-normally distributed variables. Categorical variables were presented as frequencies and percentages. Between-group comparisons employed Student’s *t*-tests for normally distributed continuous variables and Mann–Whitney U tests for non-normally distributed variables. Chi-square or Fisher’s exact tests were used for categorical variable comparisons. Correlation analyses utilized Pearson’s correlation for parametric data and Spearman’s correlation for non-parametric data. Statistical significance was set at *p* < 0.05 for all analyses. Linear regression analysis was performed to assess the association between independent variables and the dependent variable, with results reported as unstandardized coefficients (β) and significance values (*p*). Effect sizes for continuous variables were calculated using Cohen’s d, interpreted as small (0.2), medium (0.5), and large (0.8). For categorical variables, effect sizes were reported as Cramer’s V, with values of approximately 0.1, 0.3, and 0.5 representing small, medium, and large effects, respectively.

## 3. Results

### 3.1. Participant Characteristics

**Figure 1:**
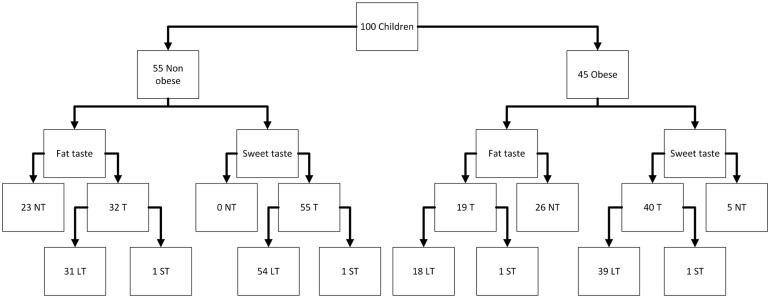
Flowchart of the population. NT = Non-Tasters; T = Tasters; LT = Low Tasters; ST = Super Tasters.

The study population consisted of 100 children, with a female predominance (54% vs. 46% male), resulting in a male-to-female ratio of 0.85 (Figure 1). Mean age was 8.05 ± 1.44 years (range: 6–12 years). Among participants, 45 children were classified as obese and 55 as non-obese based on the WHO BMI percentile criteria. Children with obesity demonstrated significantly higher mean weight (41.3 ± 12.7 kg vs. 26.4 ± 5.18 kg) and height (133 ± 10.3 cm vs. 128 ± 10.6 cm, *p* = 0.046) compared to non-obese participants.

### 3.2. Taste Sensitivity Distribution

The 3-AFC testing revealed that 93% of children were classified as low tasters for sweet taste, while 49% were classified as low tasters for fat taste. For the detection of sweet taste, 95% of children could perceive sweetness at some concentration level, with most requiring relatively high concentrations (typically 0.02 mol/L or greater). Fat taste perception was detected in 51% of participants, with recognition thresholds commonly occurring at concentrations of 3.00 mM or higher.

### 3.3. Gender and Age Associations

Analysis revealed a significant association between gender and fat taste sensitivity (Table 3). Girls were more likely to be classified as fat tasters, with 68.6% of girls versus 31.4% of boys in the taster group, compared to 38.8% of girls and 61.2% of boys in the non-taster group (*p* = 0.003, effect size = 0.279; small-to-moderate association). No significant gender difference was found for sweet taste perception, with 55% of sweet tasters being girls and 44% boys, compared to 20% girls and 80% boys in the non-taster group (*p* = 0.177, effect size = 0.109; small).

Age did not significantly differ between fat tasters (8.12 ± 1.45 years) and non-tasters (7.98 ± 1.44 years; *p* = 0.634, effect size = 0.097; small), nor between sweet tasters (8.07 ± 1.42 years) and non-tasters (7.60 ± 1.82 years; *p* = 0.476, effect size = 0.327; small-to-moderate).

Snacking frequency showed no significant association with taste sensitivity. For fat taste, 96.1% of tasters and 91.8% of non-tasters reported regular snacking (*p* = 0.432, effect size = 0.047; negligible). For a sweet taste, snacking was reported by 93.7% of tasters and 100% of non-tasters (*p* = 0.432; insignificant difference). Lastly, age showed no significant association with either fat taste (*p* = 0.634) or sweet taste (*p* = 0.476) sensitivity in this pediatric population (Table 3).

### 3.4. Food Consumption Patterns

Analysis of food consumption frequency revealed several significant associations with taste sensitivity status (Table 4). In the sweet taste groups, non-tasters reported more frequent consumption of table sugar compared to tasters, with 34.7% of non-tasters consuming it daily versus 15.7% of tasters (*p* = 0.047, Cramer’s V = 0.247; small-to-moderate association). No significant differences were observed for chocolate consumption (daily: 40.0% vs. 27.4%; *p* = 0.586, V = 0.103; small) or sweet drinks (daily: 0% vs. 10.5%; *p* = 0.548, V = 0.110; small).

In the fat-taste groups, non-tasters consumed cheese less frequently, with 53.1% eating it daily compared to 56.9% of tasters; however, a higher proportion of non-tasters consumed it 1–2 times per week (42.9% vs. 25.5%; *p* = 0.039, V = 0.254; small-to-moderate effect). Sour cream intake was markedly higher in non-tasters, with 75.5% consuming it 1–2 times per week compared to 43.1% of tasters (*p* = 0.004, V = 0.333; moderate). Fast food consumption was also more frequent among non-tasters, with 6.1% eating it daily, compared to none in the taster group, and 85.7% consuming it weekly, compared to 72.5% of tasters (*p* = 0.012, V = 0.297; small to moderate). Frying frequency showed no significant difference between groups, with weekly consumption reported by 85.7% of non-tasters and 86.3% of tasters (*p* = 0.197, V = 0.196; small) (Table 4).

Food preference scoring on a 0–10 scale showed that non-tasters generally rated high-fat and high-sugar foods more favorably than tasters (Table 5). In the sweet taste groups, non-tasters had significantly higher preference scores for cookies (10.0 vs. 7.23 ± 2.60; *p* < 0.001), chips (median 10 vs. 6; *p* = 0.022), and industrial juices (median 7 vs. 6; *p* = 0.035). No significant differences were found for candies, peanuts, fries, or hamburgers (*p* > 0.05). In the fat taste groups, non-tasters rated peanuts (7.29 ± 3.27 vs. 5.65 ± 3.92; *p* = 0.026), fries (8.31 ± 2.16 vs. 7.25 ± 2.83; *p* = 0.040), and hamburgers (7.59 ± 3.19 vs. 6.12 ± 3.68; *p* = 0.035) significantly higher than tasters, and also showed a notable difference for chips (8 vs. 3; *p* < 0.001). Industrial juices were rated higher by non-tasters (median 9 vs. 6; *p* = 0.071), although this difference did not reach statistical significance (Table 5).

### 3.5. Nutritional Intake Analysis

The dietary analysis indicated no significant differences in overall caloric intake between tasters and non-tasters for either sweet or fat taste. However, specific variations in nutrients were observed. In the sweet taste groups, non-tasters consumed significantly more sucrose than tasters (85.9 ± 64.9 g/d vs. 70.3 ± 62.3 g/d; *p* = 0.033, d = 0.250; small effect). For the fat taste groups, non-tasters demonstrated significantly higher magnesium (425 ± 414 mg/d vs. 287 ± 60.8 mg/d; *p* = 0.026, d = 0.471; moderate effect) and fiber intake (22.9 ± 7.51 g/d vs. 20.3 ± 5.32 g/d; *p* = 0.048, d = 0.401; small-to-moderate effect) compared to tasters. All other macronutrient and micronutrient intakes showed no significant differences (*p* > 0.05) and negligible to small effect sizes (d = 0.038–0.687), with the largest being for vitamin C intake in the sweet taste groups (d = 0.687; moderate effect), despite the non-significance. Similarly, in the fat taste groups, other nutrient intakes were not significantly different, with effect sizes ranging from negligible (d = 0.051) to moderate (d = 0.398 for vitamin C) (Table 6).

### 3.6. Obesity and Fat Taste Perception

**Figure 2:**
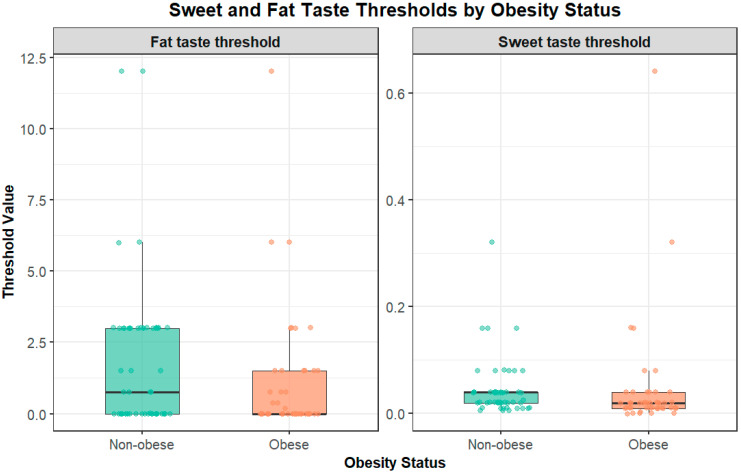
Association between fat taste/sweet perception and obesity status. Data are presented as median and interquartile range; dots represent individual values. Boxplots show fat taste thresholds (**left**) and sweet taste thresholds (**right**) in children with obesity and without obesity. Children with obesity exhibited significantly higher fat taste detection thresholds compared to children without obesity (*p* = 0.012), indicating reduced fat taste sensitivity. No significant association was observed for sweet taste thresholds (*p* = 0.731).

Children with obesity demonstrated significantly higher fat taste detection thresholds compared to children without obesity (*p* = 0.012) (Figure 2). The threshold range for children with obesity extended from 0.37 to 12.0 mM. In comparison, non-obese children showed thresholds ranging from 0.018 to 6.0 mM, indicating reduced fat taste sensitivity in the children with obesity group.

### 3.7. Obesity and Sweet Taste Perception

No significant association was observed between obesity status and sweet taste perception thresholds (*p* = 0.731) (Figure 2). The minimum recognition threshold for sweet taste was 0.005 mol/L in children with obesity compared to 0.0025 mol/L in non-obese children. Maximum recognition thresholds were higher in non-obese children (0.64 mol/L) than in children with obesity (0.32 mol/L), but these differences did not reach statistical significance.

## 4. Discussion

This cross-sectional study of 100 Tunisian children aged 6–12 years revealed several significant findings regarding taste perception and its relationship to childhood obesity. The majority of children demonstrated high recognition thresholds for both sweet (93% low tasters) and fat (49% low tasters) tastes, suggesting generally reduced taste sensitivity in this pediatric population. Girls showed significantly higher fat taste sensitivity compared to boys, while no gender differences emerged for sweet taste perception. Most significantly, children with obesity exhibited substantially higher fat taste recognition thresholds, indicating reduced fat taste sensitivity associated with increased body weight. In contrast, sweet taste perception showed no significant association with obesity status.

### 4.1. Individual Result Interpretation

#### 4.1.1. Gender Differences in Fat Taste Sensitivity

The observed female advantage in fat taste sensitivity aligns with previous research demonstrating sex-related differences in gustatory perception [16,24,31]. This finding partially supports the work of Joseph et al., who reported that girls generally demonstrate higher gustatory perception capabilities compared to boys across multiple taste modalities [13,24]. The biological basis for enhanced female fat taste sensitivity may involve hormonal influences on taste receptor expression and function, particularly during childhood development when hormonal patterns begin to establish [32]. From a clinical perspective, this gender difference suggests that boys may be at higher risk for overconsumption of high-fat foods due to reduced sensory feedback, potentially contributing to differential obesity risk patterns observed between sexes [33]. However, methodological considerations must be acknowledged, as our study did not control for pubertal status or hormonal levels, which could influence taste sensitivity during this developmental period [16].

#### 4.1.2. High Prevalence of Low Taste Sensitivity

The finding that 93% of children were classified as low tasters for sweet taste and 49% for fat taste suggests widespread reduced taste sensitivity in our pediatric population. This observation is consistent with previous research by Vennerod et al., who reported that approximately 75% of children exhibited low sensitivity to sweet taste compared to adult populations [26,27]. Similarly, studies examining fat taste perception have noted that many children require high concentrations for taste recognition, even among those classified as tasters [27,28,34]. The clinical significance of this finding is relevant, as reduced taste sensitivity may predispose children to seek higher concentrations of sweet and fatty foods to achieve satisfactory sensory experiences; however, this relationship is not straightforward, since previous studies have shown that reduced sucrose sensitivity in children does not necessarily correlate with their preferred sucrose concentration [19]. From a mechanistic perspective, this widespread hyposensitivity could result from chronic exposure to processed foods high in added sugars and fats, leading to sensory adaptation and elevated recognition thresholds [15,18]. Public health implications include the need for dietary modification strategies that account for reduced taste sensitivity when developing interventions for childhood nutrition.

### 4.2. Fat Taste Sensitivity and Childhood Obesity

The most clinically significant finding was the strong association between childhood obesity and reduced fat taste sensitivity, with children with obesity requiring substantially higher linoleic acid concentrations for taste recognition. This finding aligns with emerging research suggesting that obesity-related inflammatory processes may directly impair taste bud function and regeneration [35]. Studies by Kaufman et al. demonstrated that inflammatory cytokines associated with obesity can reduce the abundance of taste buds and inhibit cellular renewal processes, providing a biological mechanism for our observed findings [8,16]. The clinical implications are profound, as reduced fat taste sensitivity may contribute to overconsumption of high-fat foods through impaired satiety signaling and reduced sensory satisfaction [36]. This creates a potentially vicious cycle where obesity-induced taste impairment promotes continued overconsumption of obesogenic foods, further exacerbating weight gain. From a therapeutic perspective, these findings suggest that taste rehabilitation strategies may serve as novel adjunctive treatments for managing childhood obesity.

### 4.3. Absence of Sweet Taste–Obesity Association

The lack of association between sweet taste sensitivity and childhood obesity was initially unexpected, given that some previous studies had suggested altered sweet perception in obese populations [37]. However, this finding aligns with other research that has reported no significant differences in sweet taste sensitivity between obese and non-obese children [38], as well as similar results in adult populations [39,40]. Therefore, there is a heterogeneity of findings in the literature, which includes both associations and non-associations between sweet taste sensitivity and obesity.

Methodological factors may contribute to this variability, including differences in the ranges of sucrose concentrations, testing protocols, and population characteristics. Additionally, the relationship between sweet taste perception and obesity may be more complex than simple threshold detection, potentially involving preference ratings, intensity perception, or hedonistic responses rather than detection sensitivity alone [41]. The clinical interpretation suggests that fat taste sensitivity may be a more relevant biomarker for obesity risk assessment than sweet taste recognition thresholds in pediatric populations.

### 4.4. Food Consumption and Preference Patterns

The observed associations between taste sensitivity status and food consumption patterns provide essential insights into the behavioral implications of altered gustatory function. Non-tasters for both sweet and fat tastes demonstrated higher consumption frequencies and preference ratings for corresponding high-energy foods, supporting the hypothesis that reduced taste sensitivity promotes overconsumption behaviors [9]. These findings align with research by Lanfer et al., who reported that children with strong preferences for added fats or sugars had higher risks of overweight and obesity [9,23,42]. The clinical significance extends beyond simple dietary counseling, suggesting that taste sensitivity assessment could inform personalized nutrition interventions tailored to individual gustatory profiles. However, methodological limitations must be considered, as our food frequency and preference assessments relied on self and parents’ report data, which may be subject to recall bias and social desirability effects.

### 4.5. Nutritional Intake Implications

The nutritional analysis revealed several unexpected findings, including higher fiber and magnesium intake among non-tasters for fat taste, which contrasts with the general pattern of less healthy food choices in this group. Several factors may help explain this outcome. First, dietary recall methods are subject to reporting biases, and children (with parental input) may overreport foods perceived as “healthy,” such as fruits, vegetables, and whole grains, which are rich in both fiber and magnesium. Second, cultural and contextual aspects of Tunisian diets may have influenced the results: traditional meals rich in legumes, whole grains, and vegetables can simultaneously provide higher fiber and magnesium while still including fried or fatty foods.

The significantly higher sucrose intake among sweet taste non-tasters supports the primary hypothesis that reduced taste sensitivity promotes increased consumption of corresponding nutrients [15]. From a clinical nutrition perspective, these findings suggest that standard dietary recommendations may need to be modified for children with documented alterations in taste sensitivity. The practical implications include the potential need for more intensive nutritional counseling and monitoring in children identified as having reduced taste sensitivity.

The relationship between taste and the intake of essential nutrients such as vitamin C, calcium, vitamin D, and sodium in children is complex and multifaceted. High intake of sugar-sweetened beverages is associated with a decrease in the intake of essential nutrients. Specifically, children with higher intakes of sugar-sweetened beverages tend to have lower daily intakes of calcium and vitamin C [43]. Preferences for high-fat foods do not directly correlate with intake of calcium and vitamin D. However, dietary patterns rich in fats and sugars are generally associated with poor overall dietary quality, which can indirectly affect the intake of these nutrients [44]. Children who prefer high-fat foods may also consume more savory and processed foods, which are typically high in sodium. This is supported by findings that children with a preference for savory fats (e.g., pizza) have higher sodium intake [45].

### 4.6. Practical Implications and Applications

#### 4.6.1. Clinical Practice Recommendations

Healthcare providers could consider incorporating a simple 10-min fat taste screening test during routine pediatric check-ups using standardized linoleic acid solutions. Research has shown that children with reduced fat taste sensitivity often require significantly higher concentrations to detect fats and may be at increased risk of obesity. While this association is noteworthy, it is essential to emphasize that current evidence does not establish a causal relationship between fat taste sensitivity and obesity. Therefore, while such screening may help identify children who could benefit from tailored nutritional guidance, it should be viewed as a complementary tool to other methods. Pediatric dietitians should be aware that traditional dietary counseling may be less effective in children with fat taste deficits, and alternative strategies—such as texture-based and visual cue training—may be more appropriate.

#### 4.6.2. Future Research Directions

Longitudinal studies are necessary to determine whether taste rehabilitation through sensory training can restore fat taste sensitivity and prevent obesity from worsening, as this could lead to the development of the first taste-based treatments for childhood obesity. Genetic screening for taste receptor polymorphisms, combined with taste sensitivity testing, could lead to the development of personalized obesity risk profiles, enabling precision medicine in pediatric weight management. Research into taste-enhancing food technologies specifically designed for children with taste impairments could transform the food industry’s approach to developing healthy products, creating genuinely appealing and nutritious foods for high-risk groups.

### 4.7. Study Limitations

Several limitations should be considered when interpreting these findings. First, the cross-sectional design limits our ability to establish causal relationships between taste sensitivity and obesity, preventing us from determining whether reduced taste sensitivity contributes to the development of obesity or is a consequence of obesity-related changes. Second, the convenience sampling method and recruitment from a single center may limit the applicability of the results to broader pediatric populations, particularly those from diverse cultural or socioeconomic backgrounds. Third, the binary classification system (tasters vs. non-tasters) may oversimplify the complex spectrum of taste sensitivity, possibly overlooking subtle yet clinically essential variations in gustatory function. Fourth, the study did not account for potential genetic factors, such as polymorphisms in taste receptor genes, that could influence taste sensitivity and confound the observed associations. Fifth, children at this age may have limited culinary awareness; this potential misclassification, despite parental support, may represent a limitation of the study.

## 5. Conclusions

This pilot exploratory study in Tunisian children suggests that reduced fat taste sensitivity may represent a potential, easily measurable marker for risk of childhood obesity. In our sample, children with obesity required substantially higher fat concentrations to detect taste compared to their peers. The possible implications are noteworthy. If validated, simple taste sensitivity tests could be integrated into pediatric check-ups to help identify children at risk of obesity at an earlier stage, thereby informing the development of tailored behavioral and dietary interventions. Furthermore, understanding that a subset of children may have biological taste deficits could guide the design of nutrition education strategies that rely less on taste cues and more on visual, textural, and contextual approaches. At the school and community levels, enhancing the flavor of healthy foods with herbs and spices might increase their appeal for children with reduced fat taste sensitivity.

## Figures and Tables

**Table 1:** Fat taste sensitivity thresholds by taster category.

Subjects		Tested Concentration (mM)	Preparation of the Solution
Tasters	Super tasters	0.018	4 mL of the 0.18 mM solution + 20 mL of water (5% gum arabic)
		0.18	4 mL of the 0.37 mM solution + 4 mL of water (5% gum arabic)
	Low tasters	0.37	4 mL of the 0.75 mM solution + 4 mL of water (5% gum arabic)
		0.75	4 mL of the 1.5 mM solution + 4 mL of water (5% gum arabic)
		1.5	4 mL of the 3 mM solution + 4 mL of water (5% gum arabic)
		3	4 mL of the 6 mM solution + 4 mL of water (5% gum arabic)
		6	4 mL of the 12 mM solution + 4 mL of water (5% gum arabic)
		12	30 µL of the solution (3.2 M) + 8 mL of water (5% gum arabic)
Non-tasters		0	

**Table 2:** Sweet taste sensitivity thresholds by taster category.

Subjects		Tested Concentration (mol/L)	Preparation of the Solution
Tasters	Super tasters	0.00125	7.5 mL of the solution 2.5 × 10^−3^ mol/L + 7.5 mL of water
		0.0025	7.5 mL of the 5 × 10^−3^ mol/L solution + 7.5 mL of water
	Low tasters	0.005	7.5 mL of the 1 × 10^−2^ mol/L solution + 7.5 mL of water
		0.01	7.5 mL of the solution 2 × 10^−2^ mol/L + 7.5 mL of water
		0.02	7.5 mL of the solution 4 × 10^−2^ mol/L + 7.5 mL of water
		0.04	7.5 mL of the 8 × 10^−2^ mol/L solution + 7.5 mL of water
		0.08	7.5 mL of the 1.6 × 10^−1^ mol/L solution + 7.5 mL of water
		0.16	7.5 mL of the solution 3.2× 10^−1^ mol/L + 7.5 mL of water
		0.32	7.5 mL of the solution 6.4 × 10^−1^ mol/L + 7.5 mL of water
Non-tasters		0	

**Table 3:** Perception of sweet and fat tastes according to age, sex, and snacking frequency.

	Fat Taste n (%)			Sweet Taste n (%)		
	Tasters n = 51	Non-Tasters n = 49	*p*	Tasters n = 95	Non-Tasters n = 5	*p*
Age (years)	8.12 ± 1.45	7.98 ± 1.44	0.634	8.07 ± 1.42	7.60 ± 1.82	0.476
Sex female/male (%)	68.6/31.4	38.8/61.2	0.003	55/44	20/80	0.177
Snacking n (%)	49 (96.1)	45 (91.8)	0.432	89 (93.7)	5 (100)	0.432

**Table 4:** Frequency of consumption of certain foods and the perception of sweet and fat taste.

	Sweet Taste	n (%)			
Food		Every Day	1 or Twice/Week	Rarely	*p*
Chocolate	Tasters	26 (27.4)	54 (56.8)	15 (15.8)	0.586
	Non-tasters	2 (40)	3 (60)	0 (0)	
Sweet drinks	Tasters	10 (10.5)	54 (56.8)	31 (32.6)	0.548
	Non-tasters	0 (0)	4 (80)	1 (20)	
Sugars	Tasters	8 (15.7)	12 (23.5)	31 (60.8)	0.047
	Non-tasters	17 (34.7)	13 (26.5)	19 (38.8)	
	**Fat taste**	**n (%)**			
Cheese	Tasters	29 (56.9)	13 (25.5)	9 (17.6)	0.039
	Non-tasters	26 (53.1)	21 (42.9)	2 (4.1)	
Sour cream	Tasters	3 (5.9)	22 (43.1)	26 (51.0)	0.004
	Non-tasters	2 (4.1)	37 (75.5)	10 (20.4)	
Fast food	Tasters	0 (0.0)	37 (72.5)	14 (27.5)	0.012
	Non-tasters	3 (6.1)	42 (85.7)	4 (8.2)	
Frying	Tasters	3 (5.9)	44 (86.3)	4 (7.8)	0.197
	Non-tasters	0 (0.0)	42 (85.7)	7 (14.3)	

**Table 5:** Food preference according to taste perceptibility.

	Sweet Taste				Fat Taste			
	Non-Tasters n = 5	Tasters n = 95	*p*	Effect Size	Non-Tasters n = 49	Tasters n = 51	*p*	Effect Size
**Candies**	2 (1–9) *	5 (1–9) *	0.756	0.08	5 (1–8.50) *	5 (1–9) *	0.840	0.02
**Cookies**	10.0	7.23 ± 2.60	<0.001	1.50	7.82 ± 2.32	6.94 ± 2.81	0.094	0.34
**Chips**	10 (6.50–10) *	6 (1–8) *	0.022	−0.60	8 (0–10)	3 (0–10)	<0.001	−0.38
**Peanuts**	7.80 ± 2.59	6.37 ± 3.74	0.403	0.39	7.29 ± 3.27	5.65 ± 3.92	0.026	0.45
**Fries**	7.80 ± 2.86	7.77 ± 2.57	0.979	0.01	8.31 ± 2.16	7.25 ± 2.83	0.040	0.42
**Hamburgers**	7.00 ± 4.24	6.83 ± 3.49	0.917	0.05	7.59 ± 3.19	6.12 ± 3.68	0.035	0.43
**Industrial juices**	9 (6.50–10) *	6 (3–9) *	0.035	−0.48	7 (0–10) *	6 (0–10) *	0.071	−0.24

* Data are presented as median (range). Other values are presented as mean ± standard deviation. *p*-values calculated using statistical tests based on data distribution (Mann–Whitney U test for non-parametric data, independent *t*-test for normally distributed data).

**Table 6:** Dietary intake of the population according to their taste perception.

	Sweet Taste				Fat Taste			
	Non-Tasters n = 5	Tasters n = 95	*p*	Effect Size	Non-Tasters n = 49	Tasters n = 51	*p*	Effect Size
**Calories (Kcal/d)**	2028 ± 220	2048 ± 392	0.912	−0.05	2092 ± 408	2004 ± 361	0.254	0.23
**Carbohydrates (g/d)**	241 ± 29.2	234 ± 56.0	0.795	0.12	243 ± 56.9	227 ± 52.3	0.163	0.28
**Lipids (g/d)**	81.2 ± 15.8	86.7 ± 25.9	0.641	−0.21	88.2 ± 25.6	84.7 ± 25.4	0.497	0.14
**Proteins (g/d)**	68.8 ± 11.9	70.4 ± 14.5	0.809	−0.11	71.0 ± 16.4	69.8 ± 12.3	0.684	0.008
**Vitamin C (mg/d)**	48.7 ± 11.1	66.0 ± 25.6	0.831	0.06	66.6 ± 27	56.4 ± 24.2	0.986	0.01
**Magnesium (mg/d)**	308 ± 116	357 ± 306	0.720	−0.16	425 ± 414	287 ± 60.8	0.026	0.47
**Calcium (mg/d)**	578 ± 134	715 ± 203	0.141	−0.68	715 ± 221	701 ± 185	0.726	0.07
**Iron (mg/d)**	8.06 ± 1.47	7.89 ± 1.97	0.854	0.08	7.95 ± 1.93	7.85 ± 1.97	0.796	0.05
**Fibers (g/d)**	22.0 ± 12.7	21.6 ± 10.5	0.888	0.06	22.9 ± 7.51	20.3 ± 5.32	0.048	0.40
**Sucrose (g/d)**	85.9 ± 64.9	70.3 ± 62.3	0.033	0.18	74.9 ± 63.3	71.4 ± 66.1	0.507	−0.25
**Sodium (mg/d)**	2201 ± 1034	3056 ± 1169	0.112	0.740	3028 ± 1159	3000 ± 1197	0.906	0.024
**Vitamin D (µg/d)**	1.47 ± 0.78	8.35 ± 1.79	0.073	3.91	8.4 ± 1.5	7.9 ± 1.9	0.931	0.29

## Data Availability

The data that support the findings of this study are available from the corresponding author upon reasonable request.
